# Supplementary material for: Investigating the Effects of Gossypetin on Liver Health in Diet-Induced Pre-Diabetic Male Sprague Dawley Rats
Source: Molecules. 2025 Apr 19;30(8):1834. doi: 10.3390/molecules30081834 (PMC12029341; doi:10.3390/molecules30081834)
Supplement: Supplementary file 1 [file molecules-30-01834-s001.zip › Supplementary material/Supplementary Material S1.pdf]

Sterol regulatory element binding protein 1c (SREBP1c) ELISA kit protocol (catalog no.: EKC39725)

Available: https://www.biomatik.com/elisa-kits/rat-sterol-regulatory-element-binding-protein-1c-srebp-1c-elisa-kit-cat-ekc39725/

Plasma SREBP1c levels were measured according to the manufacturer's instructions using an ELISA kit (Biomatik USA, LLC, Wilmington, DE, USA). For each well, 100 μL of the standard or sample was added and incubated at 37°C for 2 hours. The liquid was removed without washing. Then, 100 μL of biotin-antibody (1x) was added to each well and the plate was incubated at 37°C for 1 hour. After incubation, the wells were aspirated and washed three times with wash buffer, with each wash lasting 2 minutes. The remaining wash buffer was removed and the plate was blotted on clean paper towels. Next, 100 μL of horseradish peroxidase (HRP)-avidin (1x) was added, and the plate was incubated again at 37°C for 1 hour. The washing process was repeated five times. Then, 90 μL of TMB substrate was added and the plate was incubated at 37°C for 15-30 minutes while being protected from light. Finally, 50 μL of stop solution was added and the plate was gently tapped to ensure thorough mixing. Optical density (OD) at 450 nm was determined using the Spectrostar Nanospectrophotometer (BMG Labtech, Ortenberg, Baden-Württernberg, LGBW, Germany).
