# Supplementary material for: Investigating the Effects of Gossypetin on Liver Health in Diet-Induced Pre-Diabetic Male Sprague Dawley Rats
Source: Molecules. 2025 Apr 19;30(8):1834. doi: 10.3390/molecules30081834 (PMC12029341; doi:10.3390/molecules30081834)
Supplement: Supplementary file 1 [file molecules-30-01834-s001.zip › Supplementary material/Supplementary Material S2.pdf]

Triglyceride colorimetric assay kit protocol (GPO-PAP method, catalog no.: E-BC-K238)

Available: https://www.elabscience.com/p/triglyceride-tg-colorimetric-assay-kit-single-reagent-gpo-pap-method--e-bc-k238

Liver triglyceride (TAG) levels were measured according to the manufacturer's instructions using an assay kit (Elabscience Biotechnology Co., Ltd., Houston, TX, USA). In the operating procedure, 2.5 μL of double-distilled water was added to the blank well, 2.5 μL of standard was added to the standard well and 2.5 μL of sample was added to the sample well. Each well was then filled with 250 μL of enzyme working solution and the contents were mixed thoroughly. The plate was incubated at 37°C for 10 minutes. After the incubation, the optical density (OD) was measured at 510 nm using the Spectrostar Nanospectrophotometer (BMG Labtech, Ortenberg, Baden-Württernberg, LGBW, Germany).

To calculate the triglyceride concentration in tissue and cell samples the following formula was used:

TG (mmol/g protein)= $\frac{\Delta A1 \times c \times f}{A2 \times Cpr}$

ΔA1 represents the difference between the OD of the sample and the blank (OD Standard – OD Blank). ΔA2 is the difference between the OD of the standard and the blank (OD Standard – OD Blank). The concentration of the standard is denoted by c, and the dilution factor of the sample before testing is represented by f. Finally, Cpr refers to the protein concentration in the sample (g/L).
