# Supplementary material for: Investigating the Effects of Gossypetin on Liver Health in Diet-Induced Pre-Diabetic Male Sprague Dawley Rats
Source: Molecules. 2025 Apr 19;30(8):1834. doi: 10.3390/molecules30081834 (PMC12029341; doi:10.3390/molecules30081834)
Supplement: Supplementary file 1 [file molecules-30-01834-s001.zip › Supplementary material/Supplementary Material S3.pdf]

Total superoxide dismutase (SOD) activity assay kit protocol (WST-1 method, catalog no.: E-BC-K020-M)

Available: https://www.elabscience.com/p/total-superoxide-dismutase-t-sod-activity-assay-kit-wst-1-method--e-bc-k020-m

Liver SOD activity was measured according to the manufacturer's instructions using an assay kit (Elabscience Biotechnology Co., Ltd., Houston, TX, USA). For the preparation of wells, the following steps were performed: In the control wells, 20 μL of double-distilled water and 20 μL of enzyme working solution were added. For the blank control wells, 20 μL of double-distilled water and 20 μL of enzyme diluent were added. In the sample wells, 20 μL of the sample and 20 μL of enzyme working solution were added. Next, 200 μL of the substrate application solution was added to each well using a multi-channel pipettor and mixed thoroughly. The plate was incubated at 37°C for 20 minutes. Finally, the optical density (OD) values of each well were measured at 450 nm using the Spectrostar Nanospectrophotometer (BMG Labtech, Ortenberg, Baden-Württernberg, LGBW, Germany).

For the determination of SOD activity in tissue and cell samples, the following formula was used:

$$i=\frac{(Acontrol-Ablank\ control)-(Asample-Ablank\ control)}{Acontrol-Ablank\ control} \times 100\%$$

$$\text{SOD activity (U/mg protein)} = i \div 50\% \times \frac{V1}{V2} \times f \div Cpr$$

i represents the inhibition ratio of SOD (%), V1 is the total volume of the reaction (240 μL), V2 is the volume of the sample added to the reaction (20 μL), f is the dilution factor of the sample before the test, and Cpr refers to the concentration of protein in the sample (gprot/L).
