# Supplementary material for: Investigating the Effects of Gossypetin on Liver Health in Diet-Induced Pre-Diabetic Male Sprague Dawley Rats
Source: Molecules. 2025 Apr 19;30(8):1834. doi: 10.3390/molecules30081834 (PMC12029341; doi:10.3390/molecules30081834)
Supplement: Supplementary file 1 [file molecules-30-01834-s001.zip › Supplementary material/Supplementary Material S4.pdf]

Glutathione peroxidase (GSH-Px) activity assay kit (catalog no.: E-BC-K096-S)

Available: https://www.elabscience.com/p/glutathione-peroxidase-gsh-px-activity-assay-kit--e-bc-k096-s?srsltid=AfmBOopfrZFlyHjfbyGcGpwQ10iiyR07aPAocqzGkmjnWivth0BwFzzH

Liver glutathione peroxidase (Gpx) activity was measured according to the manufacturer's instructions using an assay kit (Elabscience Biotechnology Co., Ltd., Houston, TX, USA). This protocol involves an enzymatic reaction followed by the chromogenic reaction.

In the enzymatic reaction, 0.2 mL of 1 mmol/L GSH standard solution was added to a 5 mL EP tube for the non-enzyme tube. For the enzyme tube, 0.2 mL of the GSH standard solution and 0.2 mL of sample were thoroughly mixed in the same size tube. These tubes were preheated at 37°C for 5 minutes with the stock application solution also heated under the same conditions. Thereafter, 0.1 mL of the stock application solution was added to all tubes and the reaction was carried out for 5 minutes at 37°C. For the non-enzyme tube, 2 mL of acid reagent and 0.2 mL of sample were added, while the enzyme tube received only 2 mL of acid reagent. The tubes were mixed well, centrifuged at 3100×g for 10 minutes and 1 mL of the supernatant was taken for the chromogenic reaction.

For the chromogenic reaction, 1 mL of supernatant from the non-enzyme tube was added to a 5 mL eppendorf (EP) tube, and the same volume was transferred from the enzyme tube to a separate EP tube. A blank well was prepared with 1 mL of GSH standard application solution, and the standard wells received 1 mL of 20 μmol/L GSH standard solution. Each tube was then supplemented with 1 mL of phosphate application solution, 0.25 mL of DTNB solution, and 0.05 mL of salt application solution. After thorough mixing, the tubes were incubated at room temperature for 15 minutes. The OD was measured at 412 nm using the Spectrostar Nanospectrophotometer (BMG Labtech, Ortenberg, Baden-Württernberg, LGBW, Germany).

For the determination of Gpx activity in tissue and cell samples, the following formula was used:

GSH-Px activity (U/mg protein)=$\frac{\Delta A1}{\Delta A2} \times c \times f2 \times f \div (V \times Cpr)$

$\Delta A1$ is the difference between the OD readings of the non-enzyme and enzyme tubes. $\Delta A2$ is the difference between the OD of the standard and the blank. The concentration of the standard (c) is 20 μmol/L and f is the dilution factor of the sample prior to testing.
